# Novel sustainable biobased flame retardant from functionalized vegetable oil for enhanced flame retardancy of engineering plastic

**DOI:** 10.1038/s41598-019-52039-2

**Published:** 2019-11-04

**Authors:** Boon Peng Chang, Suman Thakur, Amar K. Mohanty, Manjusri Misra

**Affiliations:** 1 Bioproducts Discovery and Development Centre, Department of Plant Agriculture, Crop Science Building, University of Guelph, 50 Stone Road East, Guelph, Ontario N1G 2W1 Canada; 2 School of Engineering, Thornbrough Building, University of Guelph, 50 Stone Road East, Guelph, Ontario N1G 2W1 Canada

**Keywords:** Polymer characterization, Materials science

## Abstract

The flame retardancy of an engineering plastic, poly(butylene terephthalate) (PBT), with a biobased flame retardant (FR) made from phosphorylated linseed oil (PLO) and phosphorylated downstream corn oil (PCO) was studied. Different phosphorus moieties were incorporated into the vegetable oil backbone through a ring-opening reaction. The chemical structure of the phosphorylated oil was confirmed by Fourier-transform infrared (FTIR) and nuclear resonance magnetic (NMR) spectroscopy. It was found that the incorporation of only 7.5 wt% of PLO was sufficient to change the UL-94 fire class of PBT from non-rating to V-0. The flame-retardancy mechanism of the PBT/PLO blends was evaluated from TGA-FTIR analysis. The combined effects of the gas phase mechanism and the dripping tendency of the blends aided to retard the flame propagation effectively. As the synthesized PLO and PCO contained high free fatty acids, the acid-ester exchange reaction occurred in the blends to form oligomers during the ignition. As a result, the blend dripped immediately and the drips carried all the heat to prevent fire. This work suggests that this sustainable biobased FR could be a desirable alternative to halogen-based FRs for PBT and other engineering polymers to develop more environmentally friendly FR products for various future applications.

## Introduction

Poly(butylene terephthalate) (PBT) is an important engineering plastic which is widely used in many structural components, such as electronic housing, insulators and auto parts. This is due to its high mechanical strength, good dimensional stability, rapid crystallization rate (desirable for injection molded parts) and high heat deflection temperature^1,2^. Unlike poly(ethylene terephthalate) (PET) and poly(trimethylene terephthalate) (PTT) which are self-extinguishable upon burning, PBT is a flammable polymer with extensive dripping upon ignition^3^ due to its long aliphatic butylene hydrocarbon group^4^. Its limiting oxygen index (LOI) is around 21.5%^4,5^ which is very close to the atmospheric oxygen concentration, therefore it is easily ignited in the presence of an ignition source. Thus, different flame retardant (FR) additives have been used with PBT and glass-filled PBT to improve its FR properties. This includes the use of hybrid metal and phosphinate salts^4,6,7^, aluminium hypophosphites (AHP)^5,8^, aryl phosphates/novolac^9^, phosphorus-intumescent FR compounds^10^, 9,10-dihydro-9-oxa-10-phosphaphenanthrene-10-oxide (DOPO)-based FRs^11^, brominated-organic compound-antimony trioxide (Sb_2_O_3_)^12,13^, and brominated compounds^14,15^.

Fire retardants (FR) are an important class of additives for many engineering plastics, to develop fire resistance plastics that can be used for electronic and automotive applications, which meet the industry fire standard requirement. However, most of the conventional FRs available in the market are still based on halogen-containing compounds. Although these FRs are very effective to impart flame retardancy in plastic materials, they generate toxic gases (hydrogen halides, HCl, HBr) and smoke upon burning. The processing and production of this material is also relatively hazardous to the worker and environment. Thus, halogen free and biobased FRs are gaining more popularity in the market in recent years due to their lower toxicity, as compared to halogen-based FRs. In addition, the banning of halogen-based substance in consumer products from government bodies is increasing the demand of halogen free FR additives. This has opened up many research possibilities to innovate new eco-friendly FRs-based additives to accommodate these market needs. Phosphorus-based compounds are known as effective and popular halogen-free FR materials, which have been widely used in many commercial FR compounds. The combustion products from polymers containing phosphorus-based FR are generally less toxic than halogen-based FR upon burning^16^. Effective FR compounds with less pollution to the environment are in high demand^17^. In aligning with the aim to obtain eco-friendly and non-toxic FRs, the exploration and development of biobased FR have been increasingly reported in recent years. Fox *et al*.^18^ developed effective biobased FRs from phosphorylated modified cellulose fibers for poly(lactic acid) (PLA) composites. They observed considerable enhancements in flame retardancy of PLA after the addition of modified cellulose. In another study reported by Gao *et al*.^19^, they found remarkable reduction in flammability of polypropylene (PP) with their synthesized biobased phytic acid FR. Other green FRs, such as protein, chitin, etc., have been highlighted and review by Sonnier *et al*.^20^.

The incorporation of renewable components into conventional polymers is one of the solutions to reduce the reliance on fossil fuel resources and the associated environmental pollution. Vegetable oil derivatives from different sustainable feedstocks such as soybean oil, castor oil, tung oil, sunflower oil, corn oil, linseed oil, etc. have received attention in recent years to develop biobased polymer products and additives for various applications due to their ample availability and low cost^21^. Some of the industrial uses of vegetable oil include plasticizers, lubricants, adhesives, surfactants, composites, paints and coatings^22–24^. Though there are many works focusing on improving the flexibility of the polymer with vegetable oils, the development of functionalized vegetable oil derivatives for FRs is relatively new. In order to achieve FRs from vegetable oil, different types of phosphorus moieties are incorporated into the vegetable oil backbone. The epoxidized vegetable oil was found to be an ideal starting material as it is easy to incorporate the phosphorus moieties by ring-opening hydrolysis reactions^25^. Heinan *et al*.^26^ synthesized phosphorylated soybean oil and incorporate it into rigid polyurethane (PU) foam. They found that the flame-retardancy of the developed PU/phosphorylated soybean oil foams were comparable to the commercially available FR PU. The functionalization of different vegetable oils with phosphorus-based compounds as FR polyols for PU foam, such as aminomethylphosphine oxides^27^ and phosphorus-modified linseed oil^28^, tung oil^29^, soybean oil, orange peel oil, and castor oil^30,31^, have also been reported. The developed phosphorylated vegetable oil (PVO) has mainly been studied as a polyol and incorporated in PU to achieve flame retardancy in PUs. However, the use of PVOs in engineering plastics has not been explored properly.

In this study, biobased FRs from epoxidized linseed oil (ELO) and downstream corn oil were synthesized by phosphorylation reactions. Epoxidized linseed oil was chosen as a starting material because it contains a high epoxy value and therefore provided a chance to incorporate high amounts of phosphorous moieties. Furthermore, the fire performances of the PBT blends with synthesized phosphorylated linseed oil (PLO) and phosphorylated downstream corn oil (PCO) were also compared. The bioethanol industry produced 3.6 billion pounds of downstream corn oil in the USA in 2017^32^. The proper utilization of downstream corn oil from the bioethanol industry to develop value added and eco-friendly products could promote better sustainable development in the future. Halogen-free FRs, like traditional phosphate-based additives, usually require large amounts in order to be effective to pass the UL-94 fire test. The use of large concentrations of FR additives can adversely affect the mechanical performance of virgin plastics and composites^33^. With the aim to avoid significant reductions in mechanical properties, this work attempted to use the minimum possible amount of synthesized biobased phosphorylated FR for the PBT blends. The different PVOs were synthesized with varying phosphorous content to examine the fire performance of the PVOs after blending.

## Results and Discussion

### Characterization of the phosphorylated vegetable oil

Acid and hydroxyl values of the phosphorylated vegetable oils (PVO) were determined by the methods described by the Association of Official Analytical Chemists (AOAC) and are summarized in Table 1. The acid value of PVO was found to increase with the concentration of H_3_PO_4_, however the hydroxyl values decreased. H_3_PO_4_ acted as both a catalyst and a reactant at low concentrations (15 wt%)^25^. The nucleophilic ring-opening reaction of epoxidized vegetable oils (EVO) took place by water molecules^25^. Therefore, at low concentration of H_3_PO_4_, PVO showed low acid values and high hydroxyl values. Perversely, H_3_PO_4_ acted as a reactant at high concentrations and directly reacted with the epoxy rings in EVO^34^. Therefore, the obtained PVO with high concentrations of H_3_PO_4_ exhibited lower hydroxyl values. Generally, ring-opening hydrolysis is a slow reaction, and has great potential to react the epoxy groups with the freshly created hydroxyl groups in EVO^34^. Based on this, phosphorylated dimers and trimers may also be produced. It was found that PCO15 had higher acid values than PLO15. This is due to the received downstream corn oil having around 14% free fatty acid content (measured by titrimetric method).

**Table 1:** Characterization of synthesized PLO and PCO.

Properties	PLO15^*^	PLO20^*^	PLO25^*^	PCO15^*^
Acid value (mg of KOH/g)	98 ± 3	112 ± 2	123 ± 4	107 ± 2
Hydroxyl value (mg of KOH/g)	118 ± 5	93 ± 2	76 ± 1	106 ± 2
Weight percentage of phosphorus calculated from ^1^H NMR (wt%)	1.1	1.7	2.2	0.9
Weight percentage of phosphorus measured by ICP-OES (wt%)	1.2	1.7	2.1	0.8

^*^The number indicates the wt% of ortho-phosphoric acid used for phosporylation reaction (with respect to the weight of EVO).

Increasing the amount of incorporated phosphoric acid in PLO and PCO was attempted. However, high concentration phosphoric acid assisted to form a gel structure due to crosslinking between oil chains. In the case of PLO, 25 wt% of phosphoric acid was the highest amount which could be incorporated in the oil backbone, whereas 15 wt% phosphoric acid was the highest amount which was able to integrate in the PCO backbone. The weight percentage of phosphorus in the phosphorylated oils was calculated from proton (^1^H) nuclear magnetic resonance (NMR) spectra of the corresponding PVO and summarized in Table 1. It is clear from the values that the degree of phosphorylation was increased with incorporation of more phosphoric acid. The weight percentage of phosphorus in the phosphorylated oils was further measured by Inductively coupled plasma optical emission spectroscopy (ICP-OES), the values are very similar to the results obtained from ^1^H NMR.

**Figure 1:**
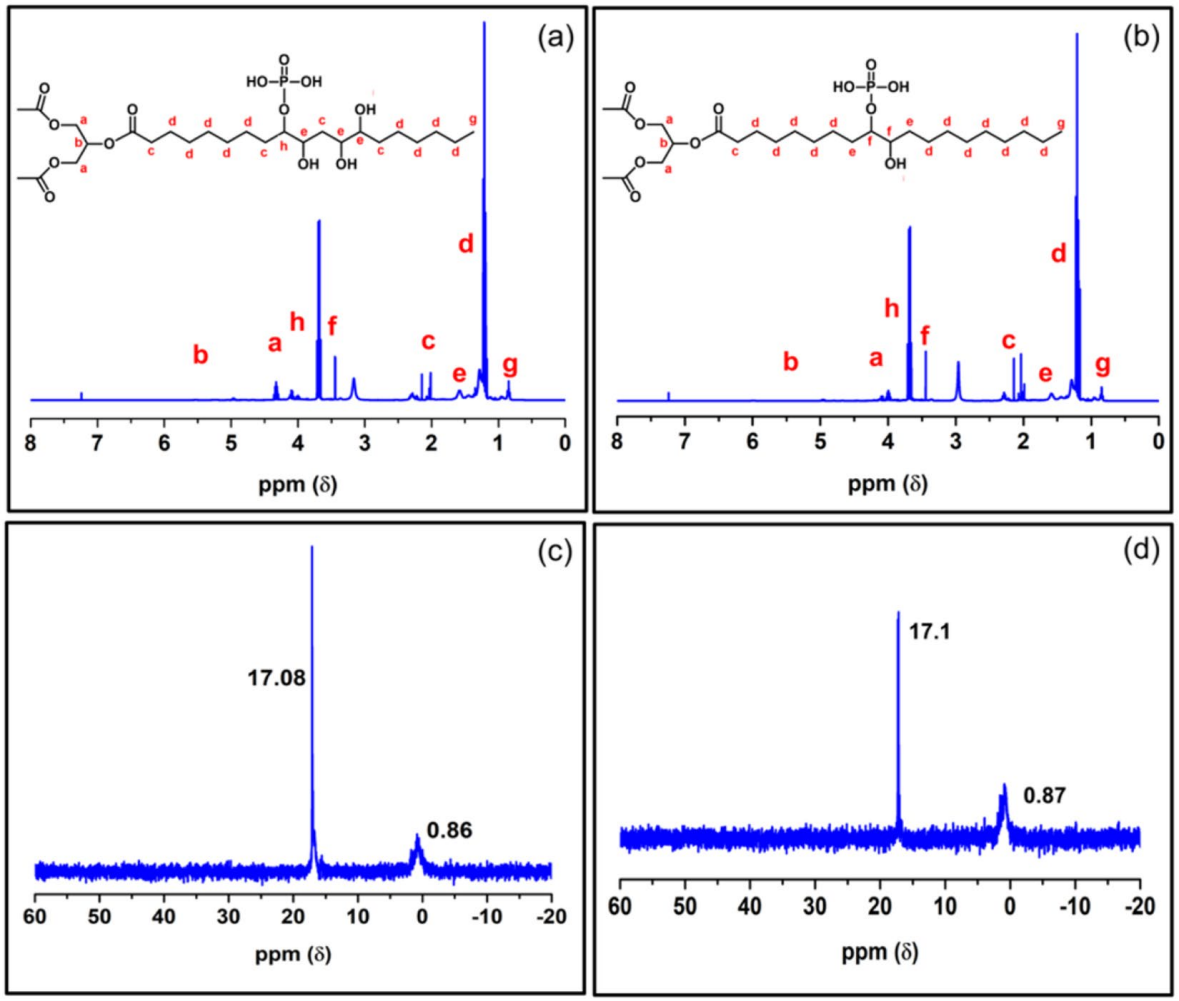
^1^H NMR spectra of (**a**) PLO15 and (**b**) PCO15; and ^31^P NMR spectra of (**c**) PLO15 and (**d**) PCO15.

NMR and Fourier-transform infrared spectroscopic (FTIR) studies were carried out to investigate the structures of the synthesized PVO. The presence of CH_2_–OC(O) and the CH–OC(O) for the inherent triglyceride structure at around 4.2 and 5.5 ppm confirmed the oil backbone in PLO15 and PCO15 (Fig. 1a,b)^34^. Proton (^1^H) NMR spectra of ELO and ECO are shown in the supporting information (Fig. S1). The presence of new peaks between 3.4 and 3.8 ppm for the protons of the carbon attached to phosphoryl groups and the disappearance of epoxy peaks in between 2.8 and 3.2 ppm confirmed the phosphorylation on ELO and ECO^26^.

**Figure 2:**
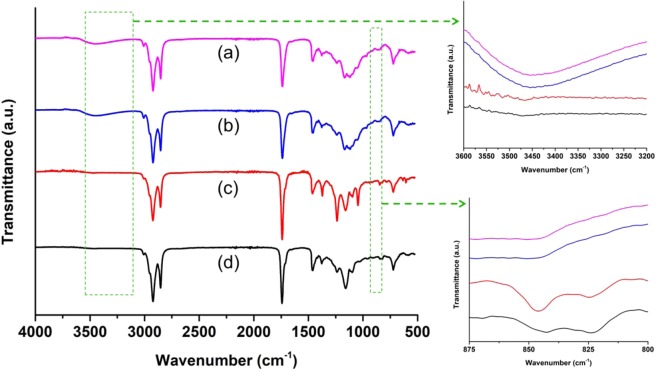
FTIR spectra of (**a**) Epoxidized linseed oil (ELO), (**b**) Epoxidized corn oil (ECO), (**c**) Phosphorylated linseed oil (PLO) and (**d**) Phosphorylated corn oil (PCO).

^31^P NMR analysis was performed to check the successful incorporation of phosphorus in PLO15 and PCO15 (Fig. 1c,d). The presence of peaks at 0.86 and 0.87 ppm for PLO15 and PCO15, respectively, indicates the formation of phosphate monoesters [ROP(O)(OH)_2_]^26^. However, there was no signal found for phosphate diesters [ROP(O)(OH)OR] or triesters [ROP(O)(OR)OR] between −2 and −20 ppm^26^. This may be due to the high amount of H_3_PO_4_ incorporation in the PLO or PCO. Both PLO15 and PCO15 showed a signal at 17.08 and 17.1 ppm, respectively for the phosphonate [R–P(O)(OH)_2_] groups. Heinen *et al*.^26^ also found similar ^31^P NMR results for phosphorylated epoxydized soybean oil. The FTIR spectra of PLO and PCO are shown in Fig. 2. The presence of new hydroxyl peaks in PLO and PCO around the 3400 cm^−1^ region and the vanished epoxy ring peak around 824 cm^−1^ suggested the ring opening of the epoxy groups^34^. After the ring opening reaction, hydroxyl groups were formed and epoxy rings were broken, which is confirmed by the FTIR spectra.

### Thermal properties

The thermogravimetric analysis (TGA) data of PBT and its blends at different ratios are shown in Table 2. The replacement of the high molecular aromatic polyester chains with vegetable oil derivatives leads to a reduction of the thermal stability of the blends, as compared to neat PBT. The onset degradation temperature (T_5%_) of the blends was decreased gradually with increased PLO content. Similar to neat PBT, only a single decomposition peak was observed for both PBT/PLO15 and PBT/PLO20 blends from room temperature to 600 °C (Fig. S2). The maximum decomposition of the blends decreased when the PLO content was further increased. For the PBT/PLO20, although the onset degradation temperature was reduced, the maximum decomposition temperature was slightly higher than in the neat PBT/PLO15. Char residue from TGA can provide information of the thermal decomposition and char formation of the samples during burning. The char residue for all the PBT/PLO blends was found to be lower than in the neat PBT (Table 2). The low char yield of the PBT/PLO blends suggested that the char formation might not be the main fire retarding mechanism. The reduction in the char yield and accelerated decomposition behavior was also reported for polycarbonate after incorporation with different amounts of phosphaphenanthrene FR^35^. The phosphorus compound presence in the PVO may have reacted with PBT, which converted it to a less flammable structure through chemical reactions or crosslinking^36^. The fire retardancy mechanism of the blends and the gas substances produced were further investigated with TGA-FTIR and will be discussed in detail in a later section. It was observed that the thermal stability of PBT/PCO blends was slightly higher than PBT/PLO blends, as shown in the TGA curves (Fig. S3). PBT/PCO blends showed approximately 10 °C higher maximum decomposition temperature than PBT/PLO blends.

**Table 2:** Thermogravimetric analysis data of PBT and its blends at different ratios.

Samples	T_(5%)_ (°C)	T_max_ (°C)	Char Residue at 600 °C (%)
Neat PBT	365.74 ± 0.80	396.59 ± 0.29	5.22 ± 1.35
PBT/PLO15(95/5)	348.85 ± 4.65	385.63 ± 4.23	1.79 ± 0.26
PBT/PLO15(92.5/7.5)	340.82 ± 1.15	379.83 ± 4.74	1.62 ± 0.59
PBT/PLO15(90/10)	341.55 ± 1.77	378.99 ± 3.95	3.65 ± 0.24
PBT/PLO20(95/5)	353.72 ± 11.11	391.06 ± 12.22	1.74 ± 0.60
PBT/PLO20(92.5/7.5)	342.13 ± 0.45	381.43 ± 0.85	1.22 ± 0.34
PBT/PLO20(90/10)	349.47 ± 2.96	394.70 ± 3.42	2.39 ± 0.33
PBT/PCO15(92.5/7.5)	342.25 ± 7.60	390.17 ± 13.92	1.74 ± 0.02

**Figure 3:**
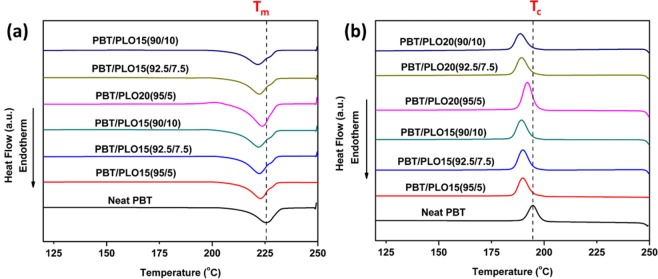
DSC (**a**) heating and (**b**) cooling curves of PBT/PLO blends with different weigh ratios.

Figure 3 presented the heating and cooling curves of the different weight ratios of PBT/PLO blends. The details of the thermal properties and degree of crystallinity are summarized in Table S1. From the DSC data, it was observed that the melting peak, *T*_*m*_, of the PBT gradually decreased with increasing PLO content for both PLO15 and PLO20. The crystallization temperature, *T*_*c*_, was shifted to a lower temperature after incorporation of PLO. The decrease in *T*_*m*_ was due to the replacement of lower molecular weight vegetable oil in the PBT. Similar observation was reported by Chieng *et al*. on the reduction of the *T*_*m*_ of PLA after incorporation of 5 wt% EVO^37^. In addition, the crystallization process of PBT was delayed with the presence of PLO, as can be seen by the reduction in *T*_*c*_. The degree of crystallinity, *X*_*c*_, was slightly reduced after incorporation of PLO. This indicates that the presence of PLO interrupted the growth of the PBT crystalline structure. The amount of PLO in the PBT matrix had a significant effect on the thermal properties, however minimal differences were seen after incorporation of PLO15 and PLO20 in the same wt%.

**Figure 4:**
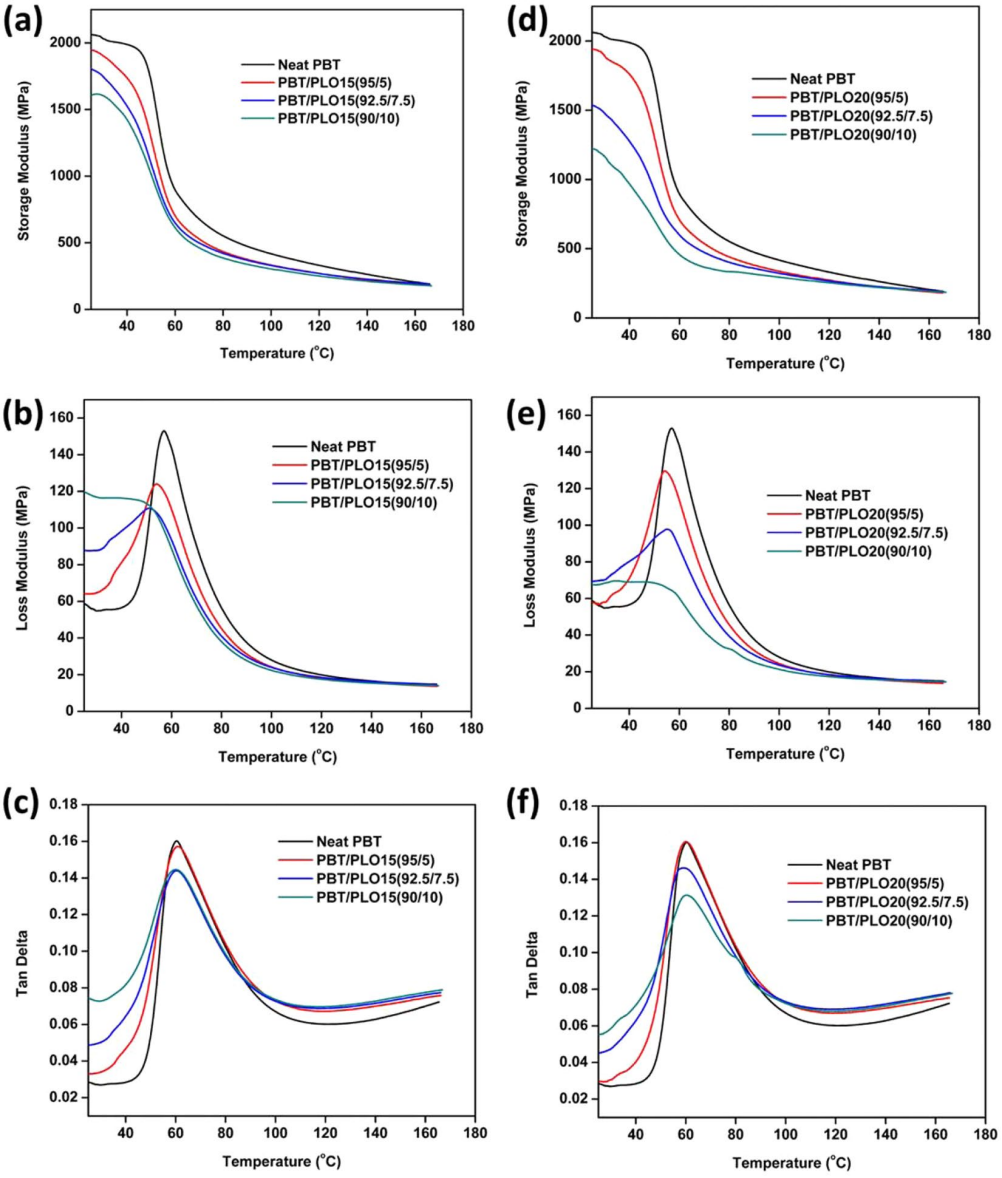
Viscoelastic behaviour of PBT/PLO blends with different weight ratios and phosphorus content. (**a**) Storage modulus, (**b**) loss modulus, and (**c**) Tan delta curves of PBT/PLO15 blends, and (**d**) storage modulus, (**e**) loss modulus, and (**f**) Tan delta curves of PBT/PLO20 blends.

The storage modulus, loss modulus and tan delta of PBT/PLO blends with different weight content are shown in Fig. 4(a–c), respectively. The storage modulus and loss modulus were decreased gradually in the blends with increasing content of PLO (Fig. 4a,b). The presence of triglyceride chains provides flexibility in the blends and acts as a plasticizer. Therefore, storage modulus and loss modulus were decreased after the incorporation of PLO. This result is well matched with other reported literature^37^. The storage modulus of PLA was found to decrease with the incorporation of epoxidized palm and soybean oil due to the plasticizing effect, which increases the chain mobility of PLA^37^.

From the tan delta curves (Fig. 4c), the damping factor of the blends decreased significantly at 7.5 wt% PLO. In addition, the glass transition temperature, *T*_*g*_, of the PBT was shifted to a lower temperature at and above 7.5 wt% loading of PLO. The presence of long triglyceride chains in linseed oil enhanced the degree of freedom of the molecular chains and free volume, which caused the shift of the PBT *T*_*g*_ to a lower temperature. The reduction in *T*_*g*_ have also been reported for other vegetable oil based polymer blends^38–40^. The chain mobility of the blends increased with increasing PLO content. Similar trends were observed for the PBT/PLO20 blends (Fig. 4d,e). It was observed that the storage modulus and loss modulus of PBT/PLO20(90/10) were much lower than in the PBT/PLO15(90/10). Oligomers may form during the processing of PBT blends by an acid-ester exchange reaction due to presence of high free fatty acid, as found in acid value of PVO (Table 1)^41^. This could attribute to the low moduli seen.

The PBT/PCO blends indicated lower storage modulus and loss modulus than PBT/PLO blends (Supporting information Fig. S4). The PCO possessed higher acid value than PLO (107 and 98 respectively at 15 wt%, refer to Table 1), so PBT/PCO blends produced more oligomers during processing than PBT/PLO blend. This contributes to the lower moduli in PBT/PCO blend.

### UL-94 horizontal and vertical burning test

Table 3 shows the UL-94 horizontal burning test results and calculated linear burning rate of the neat PBT and its blends. It was observed that neat PBT burnt readily with a ~17 mm/s burning rate upon ignition with serious flame dripping. Conversely, burning of the PBT/PLO blends was ceased immediately upon removal of the Bunsen burner after 10 s. The real time images of the samples during burning tests are presented in Fig. S5. This phenomenon was observed for all compositions of PBT/PLO blends with ratios of 95/5, 92.5/7.5 and 90/10 wt%. Hence, all the PBT blends passed the HB class for UL-94 horizontal burning test after incorporation of our synthesized FR.

**Table 3:** UL-94 horizontal burning test of the neat PBT and synthesized biobased-FR/PBT blends.

Samples	Time to extinguish (s)	Linear Burning Rate (mm/s)	Dripping	UL-94 Class
Neat PBT	Burnt completely	16.95 ± 2.52	Yes	NR
PBT/PLO15(95/5)	N/A	N/A	N/A	HB

**Figure 5:**
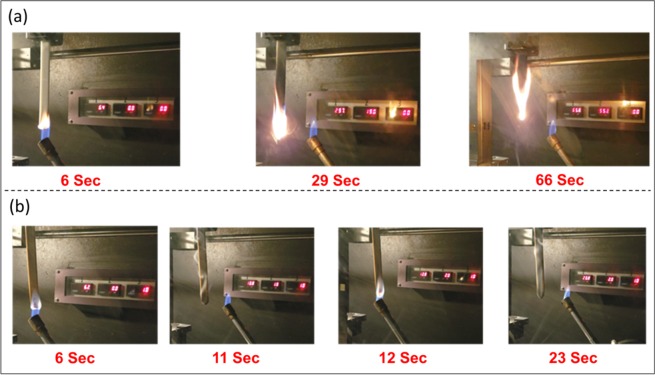
The real time image of (**a**) Neat PBT and (**b**) PBT/PLO20(92.5/7.5) blend during UL-94 vertical burning test.

In the UL-94 vertical burning test, neat PBT failed as the entire sample was burnt and fire propagated through the clamp (Fig. 5a and Table 4). Similar observations were noticed for the PBT/PLO15 and PBT/PLO20 blends at the 95/5 ratio. However, the flame retardancy of the blends was improved at the 92.5/7.5 ratio. The fire dripped during ignition and ceased immediately when the Bunsen burner was removed for both flame applications (Fig. 5b). The flame dripping was still observed during the burning and the cotton was burnt. The PBT/PLO15 blends with 7.5 and 10 wt% achieved V-2 fire class (Table 4). The fire dripping carried away the fire source quickly which helped the fire extinguish. When PLO20 and PLO25 was incorporated in the PBT matrix, the flame retardancy was further improved. With increasing phosphorus content in the vegetable oil, the flammability of the samples was reduced, as can be seen in the same loading of 95/5 for the three PBT/PLO15, PBT/PLO20 and PBT/PLO25 in Table 4. The fire class changed from NR to V-2 when synthesized PLO25 was used at 5 wt% loading in the blend. When the PLO loading increased to 7.5 wt%, all the blends achieved V-2 fire class. The photo of the remaining samples after fire tests are shown in Fig. S6. For the case of PBT/PLO20(90/10) and PBT/PLO25(92.5/7.5), the fire was difficult to ignite and was stopped immediately after removal of the Bunsen burner, therefore they obtained the UL-94 V-0 rating. For the PBT/PCO blends, V-2 fire class was also achieved. Unlike conventional flame retardant where more than 20 or 30 wt% is typically required to be effective, only 7.5 wt% of the synthesized biobased PVO is needed to achieved V-2 class in the UL-94 vertical flame test.

**Table 4:** UL-94 Vertical burning test of the neat PBT and synthesized biobased-FR/PBT blends.

Samples	1^st^ flame application (t_1_) (s)	2^nd^ flame application (t_2_) (s)	Flame dripping	Rating
Neat PBT	3.8 (0.6)	Burns completely	SDAI	NR
PBT/PLO15 (95/5)	2.3 (0.4)	Burns completely	DAI	NR
PBT/PLO20 (95/5)	3.2 (1.4)	Burns completely	DAI	NR
PBT/PLO25 (95/5)	2.4 (0.2)	10.3 (3.4)	DI	V-2
PBT/PLO15 (92.5/7.5)	2.0 (1.41)	5.75 (3.18)	DI	V-2
PBT/PLO20 (92.5/7.5)	1.3 (0.1)	2.2 (0.1)	DI	V-2
PBT/PLO25 (92.5/7.5)	1.9 (0.8)	FSAI	DI, NDAI	V-0
PBT/PCO15 (92.5/7.5)	0.95 (0.66)	2.53 (1.62)	DI	V-2
PBT/PLO15 (90/10)	1.45 (0.35)	2.55 (1.77)	DI	V-2
PBT/PLO20 (90/10)	FSAI	FSAI	DI, NDAI	V-0

*Values in table are averages with standard deviation in parentheses.

FSAI = Fire stops immediately after the removal of the ignition source;

SDAI = Severe dripping after the removal of the ignition source;

DAI = Dripping after the removal of the ignition source;

DI = Dripping during ignition;

NDAI = No dripping after the removal of the ignition source.

### Flame retardancy mechanism

**Figure 6:**
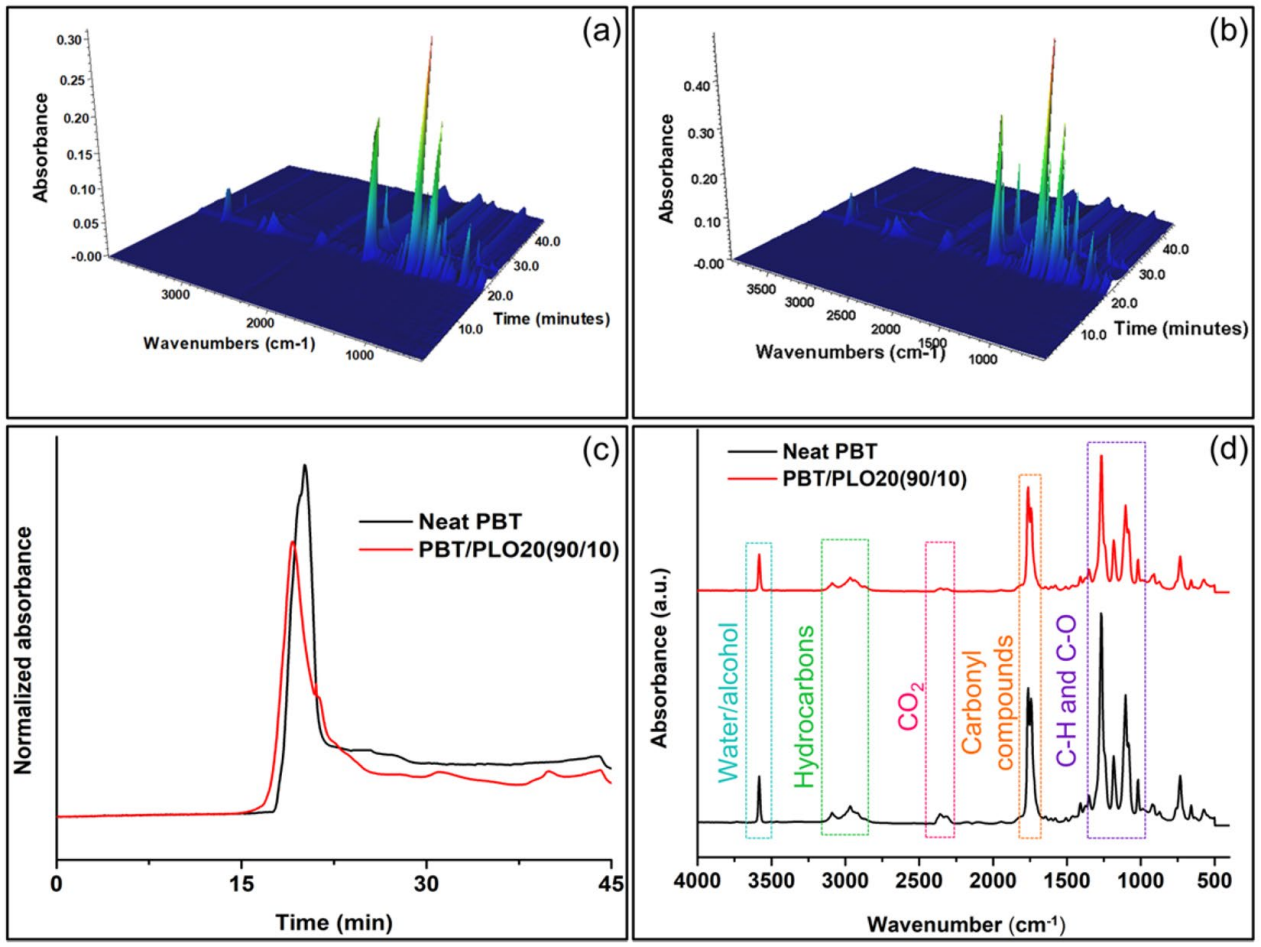
3D surface plot for TGA-FTIR spectra of the evolved gaseous products by (**a**) PBT and (**b**) PBT/PLO20(90/10) blend, (**c**) Gram−Schmidt curves PBT and PBT/PLO20(90/10) blend, and (**d**) FTIR spectra of evolved gases produced from PBT and PBT/PLO20(90/10) blend.

The gaseous products generated during thermal degradation were studied by TGA-FTIR. Three-dimensional (3D) FTIR spectra for neat PBT and the PBT/PLO20 (90/10) blend are shown in Fig. 6a,b, respectively. The total absorbance of neat PBT and the PBT/PLO20 (90/10) blend with time (Gram−Schmidt curves) are normalized by the taken mass during experiment (Fig. 6c) for proper understanding. The incorporation of PLO in the PBT blend led to a significant decrease in the intensity of Gram−Schmidt curves. This clearly indicated the presence of phosphorus containing PLO which can accelerate the reaction of radical formation, especially at high temperatures, to suppress the generation of flammable gaseous product and retard fire^42^. Zhao *et al*. found that incorporation of bis(5-formyl-2-methoxyphenyl) phenylphosphonate (BP) in PLA matrix retarded fire with a similar approach^42^. It has been reported that the thermal degradation of PVO produced gaseous phosphorus-containing products with radical such as PO^•^, PO_2_^•^, HPO_2_^•^, and HPO^•^, etc.^43^. Zhao *et al*. found the similar kind of phosphorus-containing radical products which were generated from BP to retard the flame in PLA matrix^42^. The formed phosphorus-containing radical plays crucial role. These produced radicals reacted with the H˙ and OH˙ to inhibit the radical action, in a similar action to halogen-based FR. Schartel found that H˙ and OH˙ are substituted by less active radicals or are reduced by radical recombination reaction in presence of phosphorus-containing radical^44^. Thus, the gas-phase mechanism played a crucial role in preventing burning in the PBT blends. Fig. 6d demonstrated that PBT and the PBT blend generated similar gaseous/volatile products during thermal degradations. Both the spectra showed peaks for water and alcohols (3623–3628 cm^−1^), hydrocarbons (2982–2986 cm^−1^), CO_2_ (2352–2355 cm^−1^), carbonyl compounds (1735–1746 cm^−1^), and ethers (1248–1252 cm^−1^)^45^.

**Figure 7:**
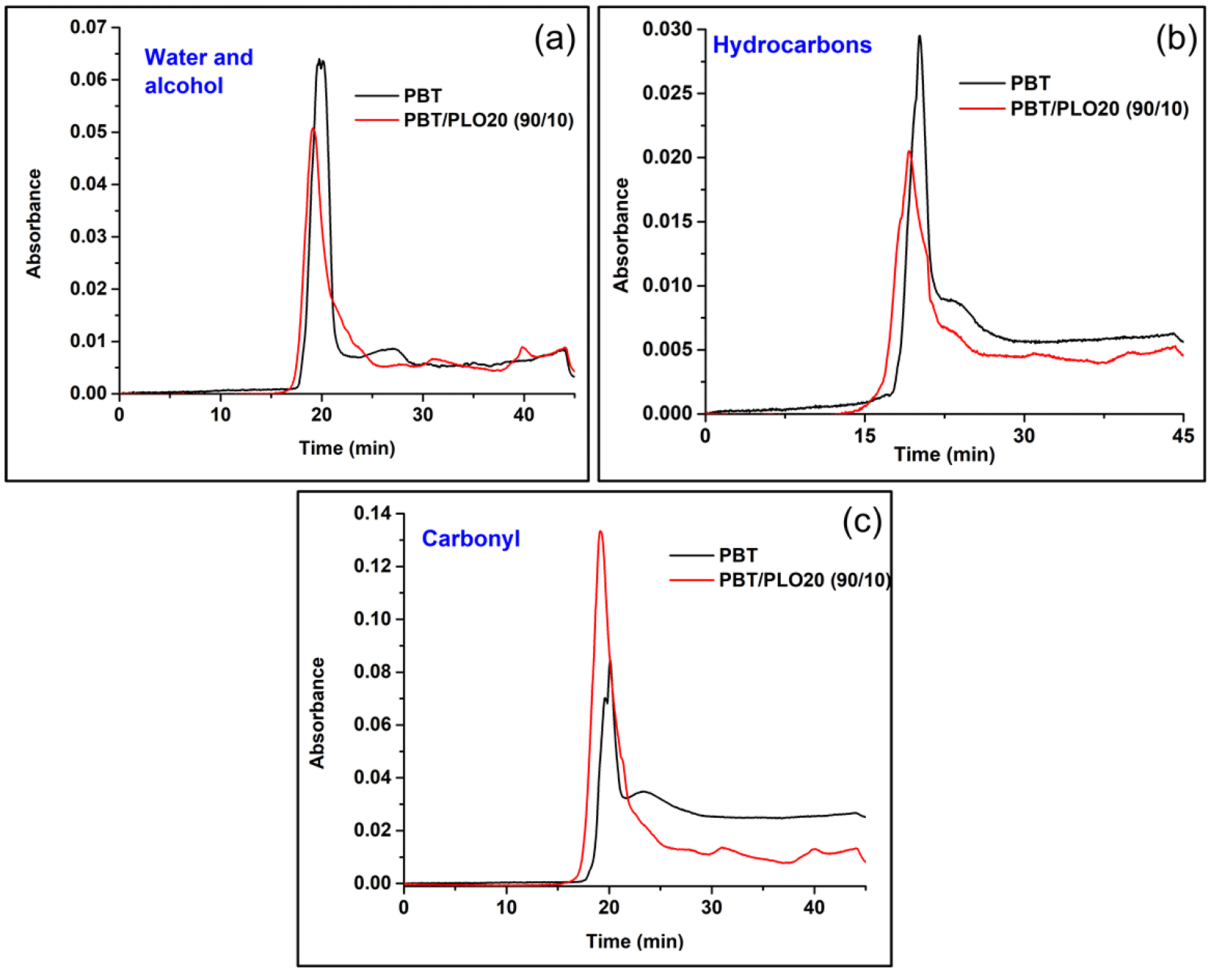
Evolved gaseous products (**a**) water and alcohols, (**b**) hydrocarbons, and (**c**) carbonyl groups containing products from neat PBT and the PBT/PLO20(90/10) blend measured by TGA-FTIR spectra.

Additionally, the normalized Gram−Schmidt curves of the carbonyl, hydrocarbons, and water and alcohol volatiles are shown in Fig. 7. The formation of hydrocarbons and water and alcohols were significantly reduced in the PBT/PLO blend (Fig. 7a,b). The decrease in the formation of hydrocarbons aided in improving the flame retardancy in the blend. However, the formation of volatile carbonyl compounds was higher in the blend. This may be due to the presence of small amounts of formed oligomers by acid-ester exchange reactions. Lin *et al*. also found similar results in case of FR unsaturated polyester resins^46^.

It is important to mention that the high acid value of PVO helped for the acid-ester exchange reaction (Table 1). This reaction resulted in the breakdown of the PBT chain and produced oligomers, which aided in easier dripping^41^. We measured the melt flow index (MFI) of the PBT and PBT/PLO blend. The MFI value of the neat PBT and PBT/PLO20(95/5) blend were 46.8 and 150.9 g/10 min, respectively. However, the MFI of the PBT/PLO20(92.5/7.5) and PBT/PLO20(90/10) could be tested in these testing conditions due to very high flowability. These blend dripped very quickly during the ignition which helped to transfer the heat away from the surface of the blend^45^. Liu *et al*. also found fast dripping aided to retard fire in polycarbonate and piperazine blends^45^. Thus, the blends effectively retarded the flame propagation due to the combined effects of the gaseous phase and dripping tendency.

### Mechanical properties and SEM morphology examination

Significant reduction in mechanical properties of polymers after incorporation of FR is a major challenge. This is due to conventional FRs requiring the addition of large amounts of FR additives in order to be effective (usually 20–30%), which results in deterioration of mechanical performance. Typically, it is very difficult to achieve UL-94 V-0 rating with low FR content in polymers^47^. Therefore, one objective of this work was to use the least amount of biobased FR to be effective as a fire retardant, without significantly reducing the mechanical performance of the polymer. Table 5 presents the tensile properties and notched impact strength of PBT/PLO blends with different blend ratios. It was observed that the tensile strength and tensile modulus of PBT reduced with increasing content of PVO. Approximately 10% reduction of tensile strength and modulus was observed for PBT/PLO15(92.5/7.5) and PBT/PLO20(92.5/7.5), as compared to neat PBT. This was due to the loss in rigidity of the PBT structures, as they were partially replaced with a more flexible aliphatic oil structure. This is confirmed with the DMA results where the storage modulus of the blends was decreased with increasing PLO content. It was also found that the modulus of the blends decreased with increasing phosphorus content in the vegetable oils. The reduction in mechanical strength and modulus of polymers after blending with vegetable oil was corroborated in several published works^37,48^. He *et al*. reported a drastic reduction in tensile strength and Izod impact strength when PBT was added with commercial FR 10-(2, 5-dihydroxyl phenyl)-9, 10-dihydro-9-oxa-10-phosphaphenanthrene-10-oxide (DOPO-HQ)^49^. They found that the DOPO-HQ required 15 wt% in the PBT in order to achieve V-2 fire class and 20 wt% to reach V-0. The tensile strength of PBT reduced ~39% from 56.9 to 34.2 MPa with the addition of 20 wt% DOPO-HQ^49^. This study’s biobased FR required only 7.5 wt% to be effective and showed only a ~13% reduction in tensile strength, which is superior to commercial phosphorus-based FRs in the market in terms of mechanical properties retention.

**Table 5:** Mechanical properties of neat PBT, PBT/PLO and PBT/PCO blends.

Samples	Tensile strength (*σt*, MPa)	Tensile modulus (*Et*, MPa)	Elongation at yield (*εY*, %)	Elongation at break (*εB*, %)	Notched impact strength, (J/m)
Neat PBT	53.6 (0.25)	2512 (321.9)	4.39 (0.21)	117.43 (57.24)	36.53 (3.63)
PBT/PLO15 (95/5)	47.6 (0.81)	2423 (26.72)	3.81 (0.09)	22.29 (3.24)	35.54 (0.00)
PBT/PLO15 (92.5/7.5)	46.8 (0.16)	2217 (139.39)	5.05 (0.38)	11.51 (4.54)	15.35 (0.91)
PBT/PLO15 (90/10)	40.3 (0.46)	1967 (80.21)	6.16 (−)	7.54 (0.73)	13.09 (1.64)
PBT/PLO20 (95/5)	52.9 (1.26)	2396 (179.26)	4.10 (0.36)	18.52 (2.19)	33.02 (0.91)
PBT/PLO20 (92.5/7.5)	46.9 (1.23)	2284 (97.73)	4.43 (0.03)	5.50 (0.83)	14.86 (1.60)
PBT/PLO20 (90/10)	39.9 (1.06)	1981 (38.89)	−	2.99 (−)	13.12 (0.02)
PBT/PLO25 (95/5)	44.0 (0.99)	2148 (112.25)	4.66 (0.24)	24.33 (1.64)	29.44 (0.93)
PBT/PLO25 (92.5/7.5)	39.2 (0.95)	1866 (49.44)	9.62 (1.86)	17.85 (1.82)	15.38 (3.18)
PBT/PCO15 (92.5/7.5)	43.0 (0.45)	2188 (33.56)	5.66 (0.46)	5.77 (0.67)	12.63 (1.55)

*Values in table are averages with standard deviation in parentheses.

The elongation at break and the notched impact strength of the PBT were also observed to decrease with increasing PLO content. The reduction in elongation and impact toughness after addition of PVO was due to the introduction of a secondary immiscible phase in the PBT. The low mechanical performance after blends indicated limited bonding and interaction between the PLO and PBT phases. PVO contains numerous functional groups which could be reacted during reactive processing, therefore a coupling reaction and compatibilization could be future steps of this work to restore the mechanical performance. The formation of oligomers by an acid-ester exchange reaction is another reason for the decreased mechanical properties. The presence of high acid value of PVO in PBT blends could have accelerated the acid-ester exchange reaction, which produced the oligomers in the blends.

**Figure 8:**
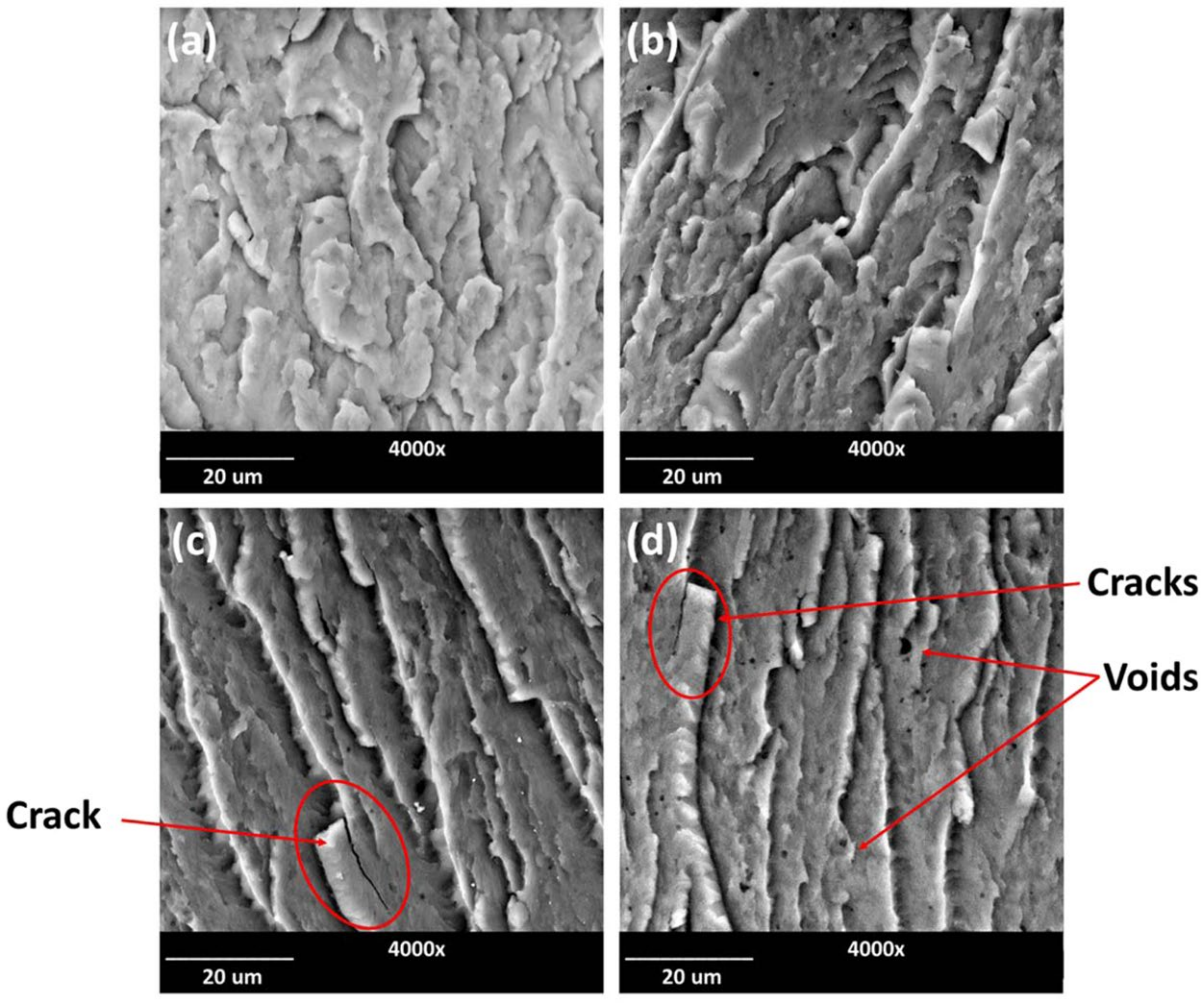
SEM micrographs of impact fractured surfaces of (**a**) Neat PBT, (**b**) PBT/PLO20(95/5), (**c**) PBT/PLO20(92.5/7.5), and PBT/PLO20(90/10) blends.

The fractured surfaces of the PBT/PLO blends were further examined with SEM (Fig. 8a–d). It was observed that the addition of PLO leads to the formation of small droplets and voids in the PBT phase due to the incompatibility between phases. The PBT exhibited brittle fracture surfaces with increasing PLO content, as can be seen by the crack formations on the fractured surfaces (Fig. 8c,d).

**Figure 9:**
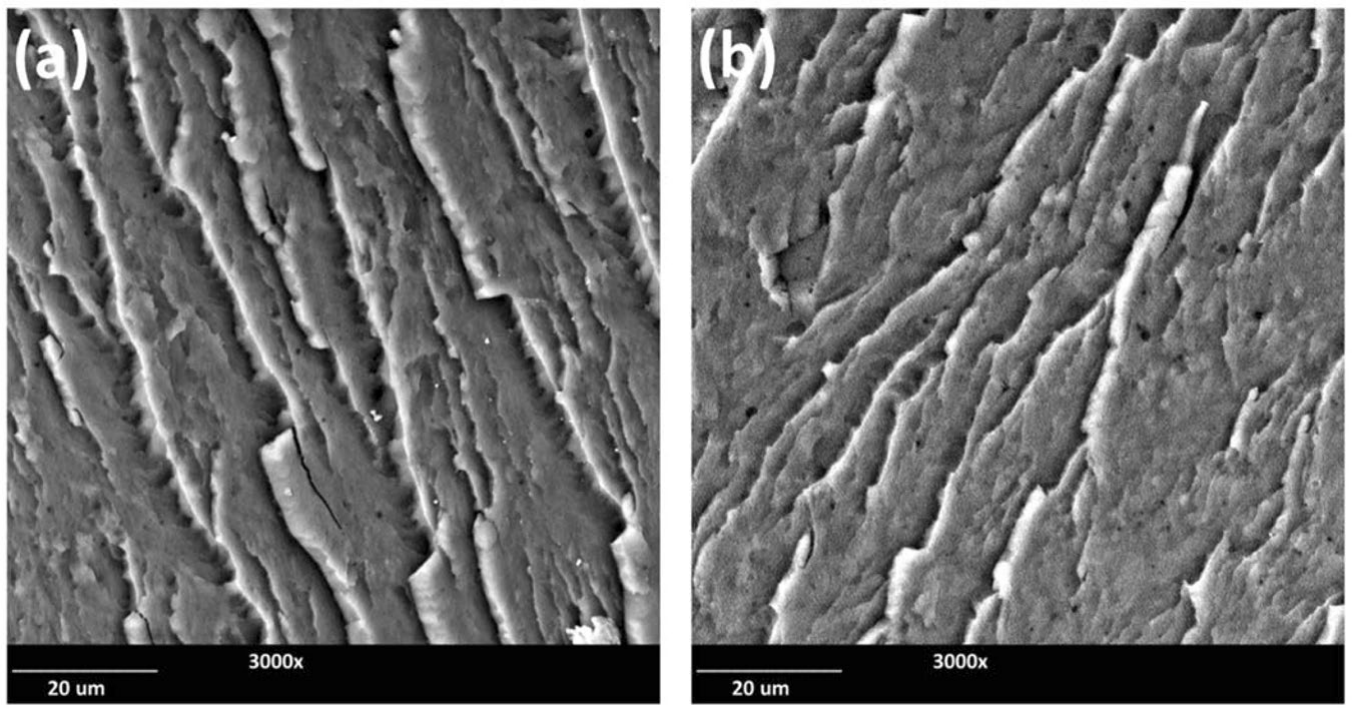
SEM micrographs of impact fractured surfaces of (**a**) PBT/PLO20(92.5/7.5) and (**b**) PBT/PCO20(92.5/7.5).

Figure 9 showed the comparison of fractured surfaces between PBT/PLO and PBT/PCO blends at the same PVO loading level. Both blends also showed brittle fracture surfaces with visible cracks and voids. However, the PBT/PCO blend showed higher amounts of voids than the PBT/PLO blend. This could be due to the higher impurity of the starting downstream corn oil, as compared to linseed oil, which have been discussed previously.

## Conclusions

Phosphorylated linseed oil (PLO) and phosphorylated corn oil (PCO) have been successfully synthesized using ring opening reactions. The flame retardancy of the engineering plastic, poly(butylene terephthalate) (PBT), was improved significantly after incorporation of the synthesized functionalized biobased flame retardant (FR) from both linseed oil and downstream corn oil. Unlike conventional FRs, the incorporation of small amounts (~10 wt%) of these biobased FR changed the UL-94 fire class of PBT from non-rating to V-0. These blends effectively retarded the flame propagation due to the combined effects of the gaseous phase and dripping tendency. The incorporation of this synthesized PLO did not compromise the mechanical performance of PBT as severely as other conventional FRs. The formation of oligomers by acid-ester exchange reaction was the main reason for the decreased mechanical properties.

Our synthesized biobased FRs from vegetable oil and downstream corn oil could advance the production of more environmentally friendly, non-halogen based FRs. These could have use in various engineering plastic products, such as electronic components and automotive parts. The utilization of waste stream corn oil from the bioethanol industry to develop a value added and eco-friendly functional product supports the circular economy model for a better future.

## Experimental Methods

### Materials

The epoxidized linseed oil (ELO) (Vikoflex 7190) was a product from Arkema, USA and downstream corn oil was kindly supplied by IGPC Ethanol, Ontario, Canada. Hydrogen peroxide (30% aqueous solution), ethyl acetate and isopropyl alcohol were received from Fisher Scientific, Canada. Formic acid (98%), sulfuric acid (98%) and ortho-phosphoric acid (85% w/w aqueous solution) were purchased from Acros Organics, Canada. Anhydrous magnesium sulfate was obtained from Sigma-Aldrich, Canada. The PBT used in this study is a product from Ticona Engineering Polymer, with the trade name of Celanex grade 2000–3 supplied by Entec Polymers, Orlando, USA. Its density was measured around 1.31 g/cm^3^.

### Synthesis of phosphorylated vegetable oil

**Scheme 1:**
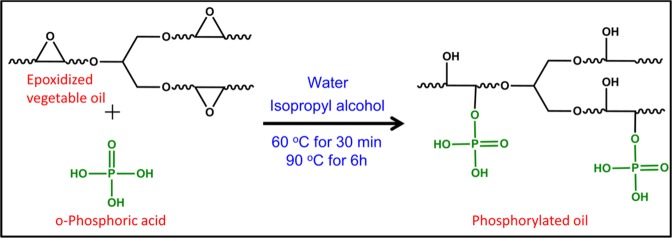
Synthesis of phosphorylated epoxidized vegetable oil.

Phosphorylated linseed oil (PLO) was synthesized by the ring-opening reaction of epoxidized linseed oil (ELO) in accordance with a reported method (Scheme 1)^34^. ELO (100 g), water (10 g, 10% by weight of ELO), and isopropyl alcohol (25 g) were taken into a three necked round bottomed flask equipped with a condenser and a thermo-sensor. The mixture was then heated at 60 °C for 30 min. Following, ortho-phosphoric acid was solubilized in 25 g of isopropyl alcohol (total added amount of isopropyl alcohol was 50% by weight of ELO) and the solution added dropwise. The temperature was raised to 90 °C after the addition of ortho-phosphoric acid, and was held for 6 h. Three different PLOs were synthesized: PLO15, PLO20 and PLO25 with 15, 20 and 25 wt% of ortho-phosphoric acid (with respect to the weight of ELO), respectively. Following the same procedure, phosphorylated downstream corn oil (PCO) was synthesized with 15 wt% of ortho-phosphoric acid (with respect to the weight of ECO) and encoded as PCO15. Epoxidized downstream corn oil (ECO) was prepared using formic acid and aqueous H_2_O_2_ solution in the presence of a catalytic amount of sulfuric acid^50^.

### Preparation of the PBT/PLO and PBT/PCO blends

The materials were dried in an oven at 80 °C overnight before processing. The neat PBT and its blends with varying amounts of PLO or PCO (5, 7.5 and 10 wt%) were melt blended in a co-rotating twin screw DSM-Xplore MC15 laboratory micro-compounder from the Netherlands. The compounding process was carried out at 250 °C for 2 min with a screw rotation of 100 rpm followed by injection molding into standard specimens for tensile, notched Izod impact and UL-94 fire tests, according to the ASTM standards. All the samples were conditioned at a temperature of 23 °C and relative humidity of 50% for at least 40 h after processing prior to testing, as according to the ASTM D618-13 (Procedure A) standard.

### Characterization

#### Nuclear magnetic resonance (NMR) spectroscopic analysis

NMR experiments were recorded in a Bruker AVANCE III spectrometer with ^1^H and ^31^P operating frequency of 600.0 MHz, using a 5 mm TCI cryoprobe. CDCl_3_ was used as a solvent for PCO and PLO. The sample temperature was kept at 22 °C for all the NMR testing.

#### Fourier-transform infrared spectroscopic (FTIR) analysis

FTIR spectra of the samples were obtained using an attenuated total reflectance (ATR) device on a Nicolet 6700 ATR-FTIR from Thermo Scientific, USA. Background was collected before capturing the spectra for each sample. The experiment was performed in the 4000–500 cm^−1^ wavenumber range with 64 scans per specimen.

#### Inductively coupled plasma optical emission spectroscopic (ICP-OES) analysis

ICP-OES analysis was carried out to determine extent of phosphorylation in PLO. For the testing, 0.1 g of the sample were microwave (CEM MARxpress) digested in closed vessels with a mixed acid (nitric acid/hydrochloric acid). The resulting digest was brought up to volume (50 mL) with Nanopure water. The solution was analyzed by ICP-OES (Agilent 5110) without further processing.

#### Thermogravimetric analysis

The thermo-degradation characteristic of the PBT/PLO and PBT/PCO blends was conducted using a TA Q500 thermal analyzer from TA-Instruments, USA. Approximately 5–8 mg of each samples were placed in a platinum pan and heated under nitrogen atmosphere with a 50 mL/min flow rate. The samples were heated from 30 to 700 °C with a heating rate of 10 °C/min. Both the TGA and the derivatives of the TGA curves (DTG) were obtained and extracted from TA universal analysis software. Two replicates were tested for each sample, and the mean and standard deviation of the results were reported.

### Thermal properties and crystallinity analysis

Thermal properties of the PBT and its blends were performed using a differential scanning calorimeter (DSC) Q200 from TA-Instruments, USA. Approximately 5–7 mg of each sample was heated from 30 to 250 °C, with a heating rate of 10 °C/min. The samples were then isothermally held for 3 min and subsequently cooled down to 30 °C at the same rate of 10 °C/min. The sample was re-heated again to 250 °C to complete the second heating process. The melting temperature (*T*_*m*_) and enthalpy of fusion (*ΔH*_*m*_) from the first heating and cooling curves of the samples from the DSC thermograms were used to analyze the thermal properties. The degree of crystallinity (*X*_*c*_) of the PBT and its blends was calculated from the ratio of area under the first melting peak of the DSC thermogram to the enthalpy of melting for 100% crystalline PBT as shown in equation (Eq. 1)

1 $${X}_{c}=\frac{\Delta {H}_{m}}{\Delta {H}_{100}(1-{w}_{f})}\times 100 \% $$

where *ΔH*_*m*_ is the enthalpy of melting for the sample, *ΔH*_100_ is the enthalpy of melting for 100% crystalline PBT (i.e. 145.0 J/g^51^) and *w*_*f*_ is the weight percent of the PLO or PCO in a particular blend.

#### Dynamic mechanical analysis

The viscoelastic behavior of the blends was investigated using a dynamic mechanical analyzer DMA Q800 from TA-Instruments, USA. Samples with dimensions of 50 mm × 12 mm × 3 mm were used in dual cantilever clamp mode at a sinusoidal stress frequency of 20 Hz and strain rate of 1%. All the samples were heated from 20 to 170 °C with a heating rate of 3 °C/min.

#### UL-94 flammability test (Underwriters Laboratories standard UL-94)

The flammability test was conducted in an R.B. Atlas HVUL2 horizontal vertical flame chamber from ATLAS Material Testing Solutions, USA according to Underwriters Laboratories (UL-94) Standard. The samples had dimensions of 120 mm × 12 mm × 3 mm (length × width × thickness) and were used for both UL-94 horizontal and vertical flammability tests, according to the ASTM D635-18 and ASTM D3801-10 respectively. For the horizontal burning test, the sample was clamped horizontally and ignited at the end tip in an oxygen atmosphere using a Bunsen burner. Fire was applied for 30 s and the time duration measurement was started after the sample burnt through 25 mm length and stopped after burning through 75 mm of the sample. The rate of burning, *V*, for each sample was calculated based on the equation (Eq. 2)

2 $$V=\frac{60L}{t}$$

where *L* is the burnt length (75 mm) and *t* is the time of burning. The reported value is an average of five samples.

For the vertical burning test, the sample was clamped vertically and ignited at the bottom end tip. The fire was removed after 10 s and the time for the fire to extinguish was recorded as after flame time t_1_. The sample was burnt for the second time for 10 s continuously after the cease of the fire, and this time was recorded as after flame time t_2_. The dripping of the samples and cotton ignition was observed and recorded.

#### Melt flow index

The melt flow index of the biobased PBT/PLO blends was carried out using a Melt Flow Indexer (Qualitest model 2000A), according to ASTM D1238 standard. All the blends were tested at a temperature of 235 °C and load of 2.16 kg.

#### TGA–FTIR analysis

The TGA–FTIR analysis were performed using a TA 5500 thermogravimetric analyzer system coupled with a Thermo Scientific Nicolet 6700 FTIR spectrometer in nitrogen at a flow rate of 50 ml/min. During the formal tests, approximately 5 ± 1 mg of each sample was heated in the TGA at a heating rate of 20 °C/min from room temperature to 900 °C.

#### Mechanical testing and fracture morphology

Tensile and notched Izod impact tests were carried out according to ASTM D638 (Type IV) and D256, respectively. The tensile test was performed using an Instron 3382 Universal Testing Machine with a crosshead speed of 5 mm/min. Notched Izod impact tests were carried out using a TMI 43-02 Monitor Impact Tester (Testing Machines Inc., New Castle, DE, USA). The samples were notched in a notch cutter following processing.

The impact fractured surfaces of the PBT/PLO and PBT/PCO blends were examined using a Phenom ProX desktop scanning electron microscope (SEM) from Phenom World, Eindhoven, Netherlands.

## Supplementary information

Electronic Supplementary Information
