# Supplementary material for: Novel sustainable biobased flame retardant from functionalized vegetable oil for enhanced flame retardancy of engineering plastic
Source: Sci Rep. 2019 Nov 4;9:15971. doi: 10.1038/s41598-019-52039-2 (PMC6828712; doi:10.1038/s41598-019-52039-2)
Supplement: Supplementary file 1 — Electronic Supplementary Information [file 41598_2019_52039_MOESM1_ESM.docx]

**Electronic Supplementary Information**

**Novel sustainable biobased flame retardant from functionalized vegetable oil for enhanced flame retardancy of engineering plastic**

Boon Peng Chang^a,^, Suman Thakur^a^, Amar K. Mohanty*^a,b,^ and Manjusri Misra^a,b^

^a^Bioproducts Discovery and Development Centre, Department of Plant Agriculture, Crop Science Building, University of Guelph, 50 Stone Road East, Guelph, Ontario, N1G 2W1, Canada.

^b^School of Engineering, Thornbrough Building, University of Guelph, 50 Stone Road East, Guelph, Ontario, N1G 2W1, Canada.


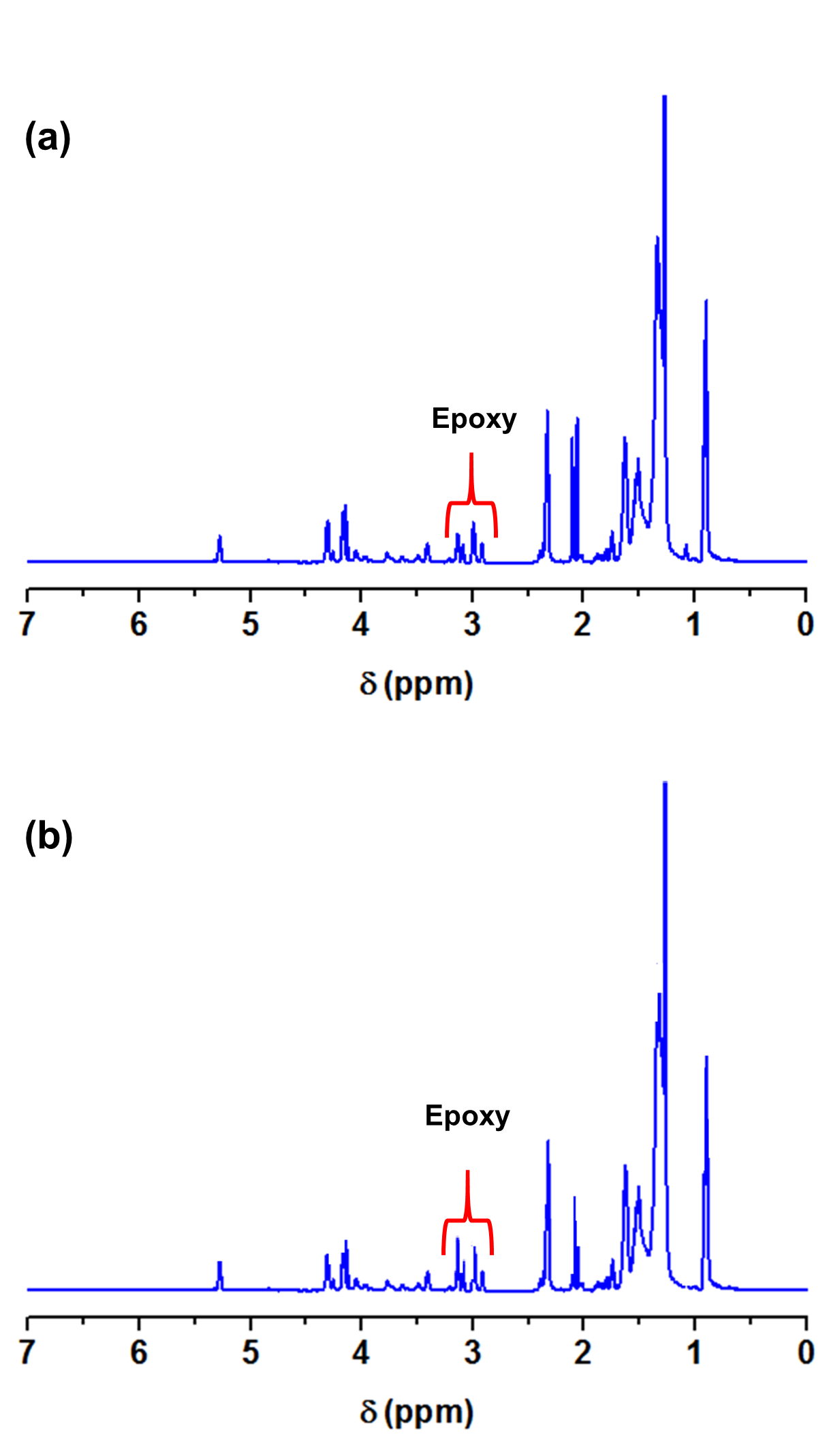


**Figure S1** ^1^H NMR spectra of (a) epoxidized downstream corn oil (ECO) and (b) epoxidized linseed oil


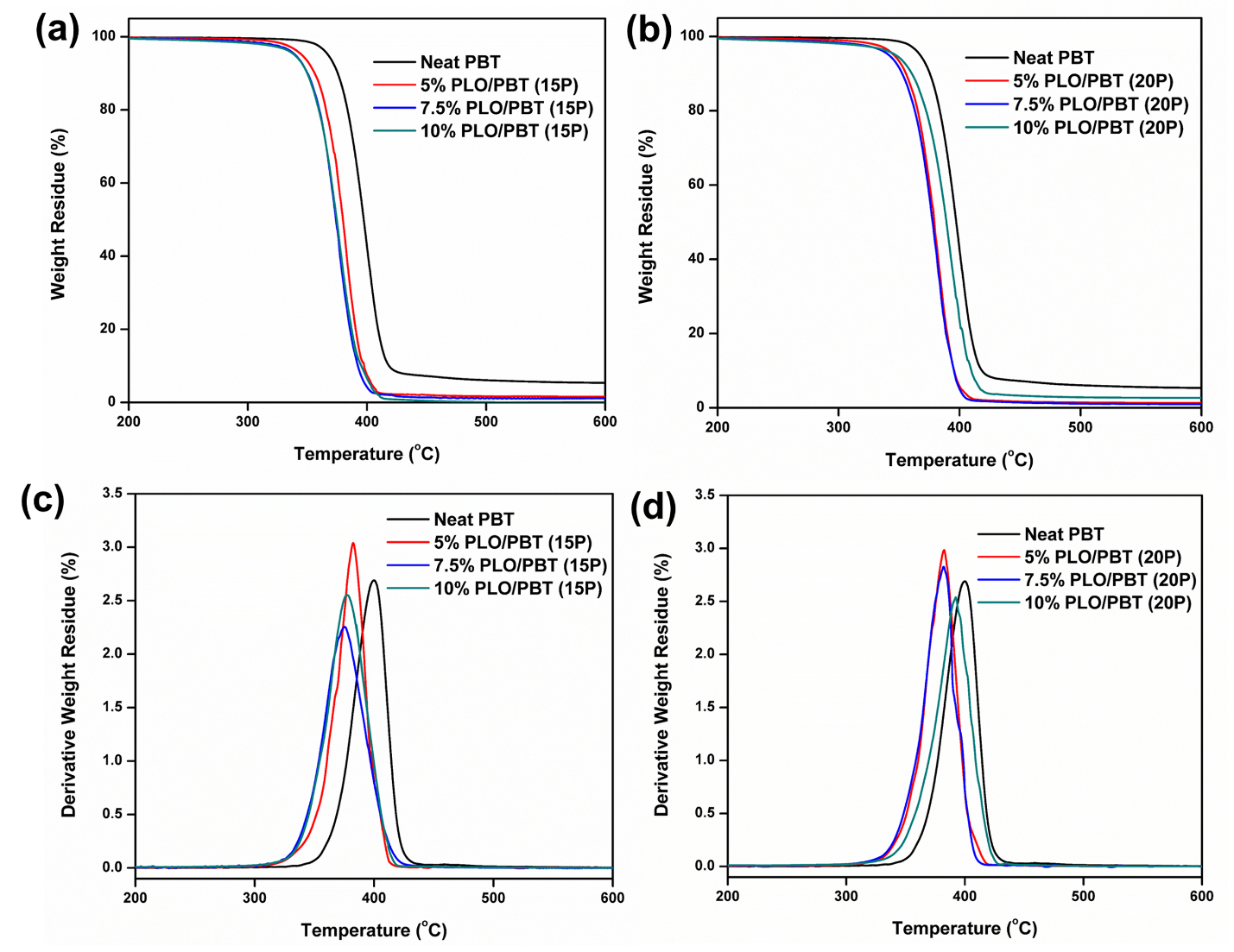


**Figure S2** Thermal degradation and derivative thermal decomposition curves of neat PBT and its blends with different PLO content for PBT/PLO15 (a, b) and PBT/PLO20 (c, d).


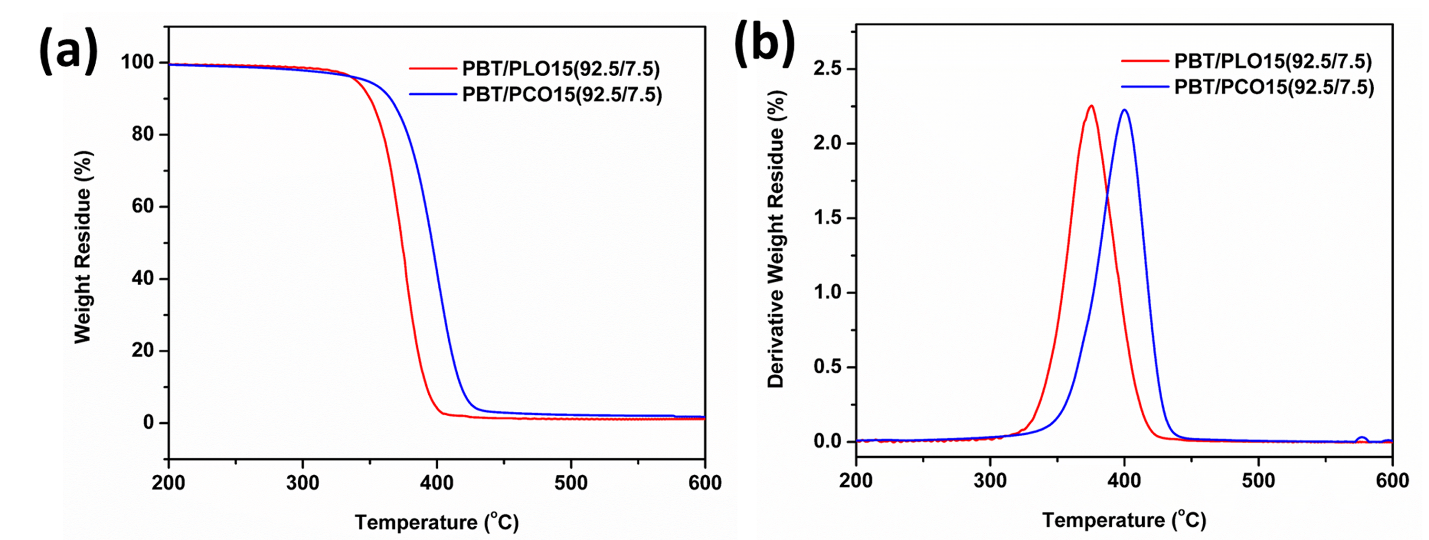


**Figure S3** Thermal degradation and derivative thermal decomposition curves of PBT/PLO15 and PBT/PCO15 blends with 92.5/7.5 blend ratio.


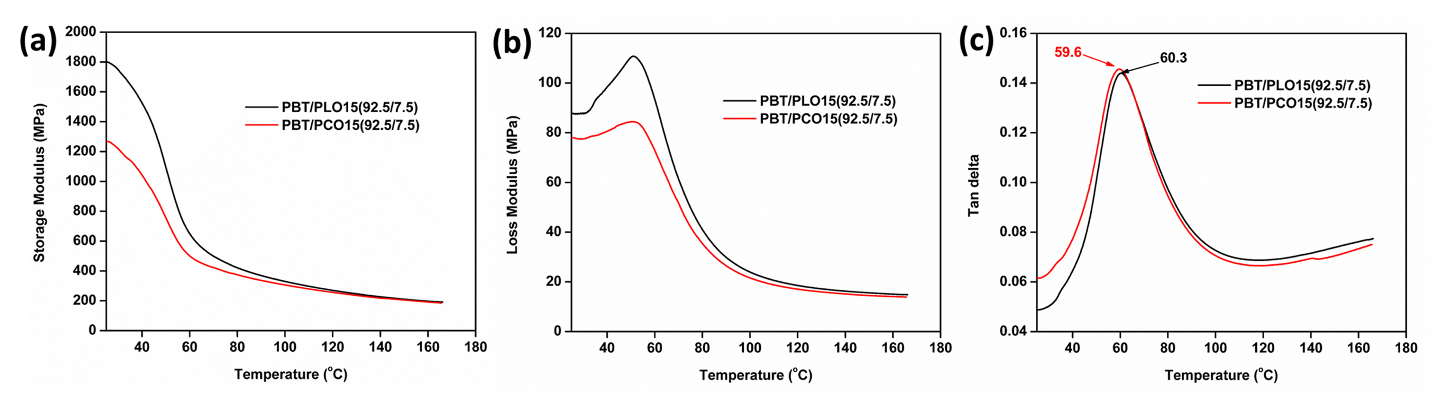


**Figure S4** Visoelastic behaviour of PBT/PLO and PBT/PCO blends at 92.5/7.5 blend ratios. (a) Storage modulus, (b) Loss modulus and (c) Tan delta curves.


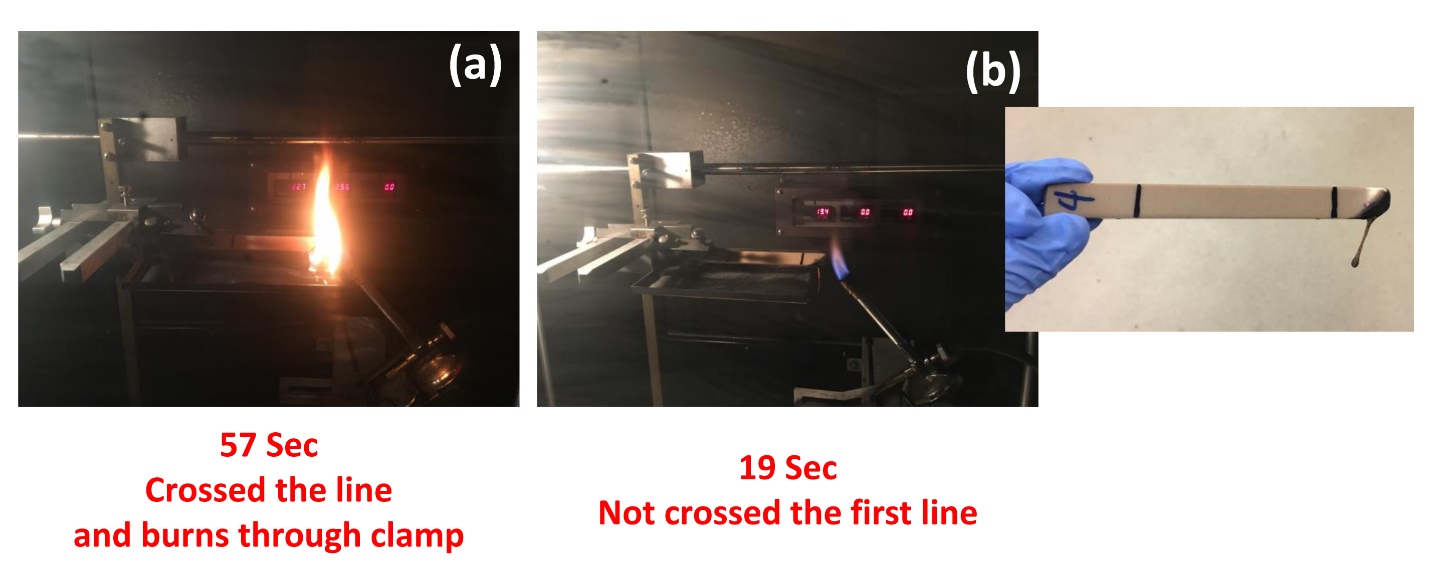


**Figure S5** The real time image of (a) Neat PBT and (b) PBT/PLO15(95/5) blend during UL-94 horizontal burning test and the inset photo showed the photo of PBT/PLO15(95/5) sample after the horizontal burning test.


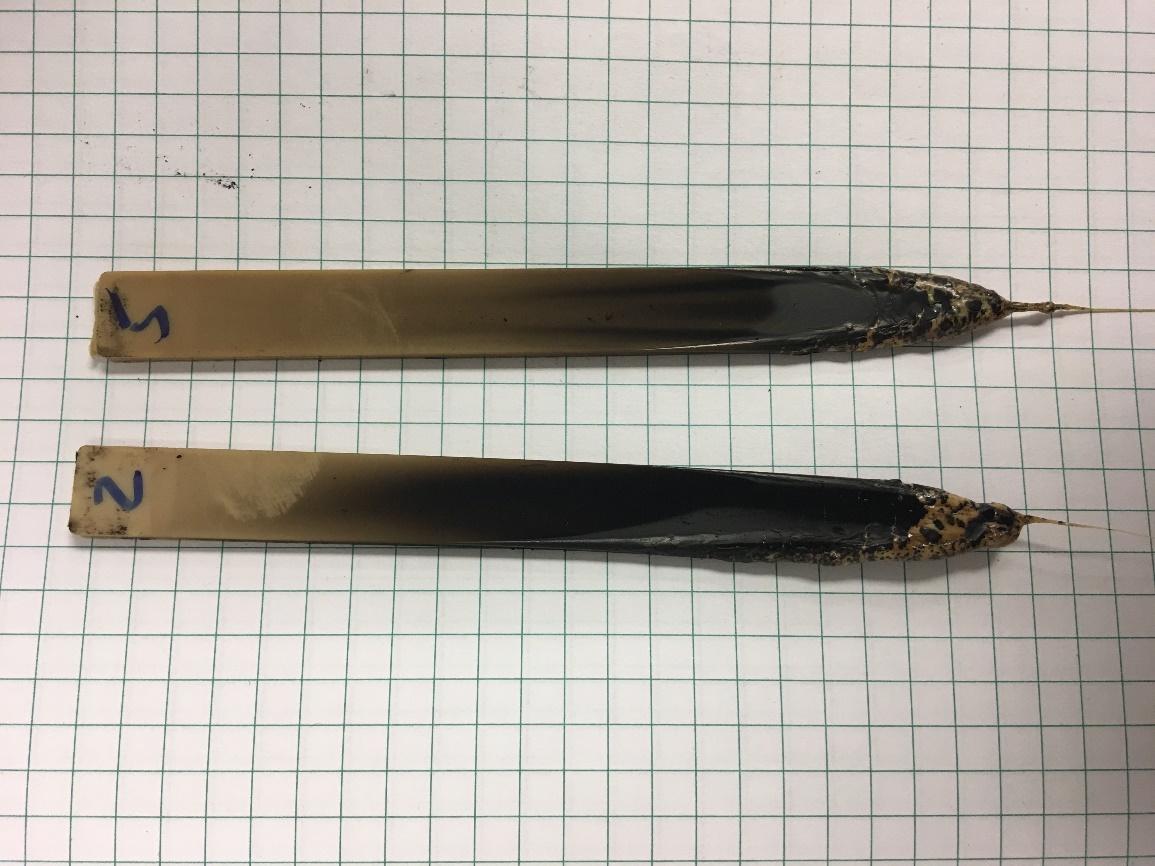


**Figure S6** Photos of the flame dripping formation and residues of the PBT/PLO blends.

**Table S1** DSC data for PBT/PLO blends with different blend ratios and phosphorus content.

| **Samples** | ***T_m_* (C)** | ***T_c_* (C)** | ***H_m_* (J/g)** | ***X_c_* (%)** |
| --- | --- | --- | --- | --- |
| Neat PBT | 226.5 ± 1.4 | 194.4 ± 0.3 | 50.7 ± 0.3 | 35.0 ± 0.2 |
| PBT/PLO15(95/5) | 223.0 ± 0.2 | 190.0 ± 0.2 | 49.7 ± 2.1 | 32.6 ± 1.4 |
| PBT/PLO15(92.5/7.5) | 222.5 ± 0.2 | 189.8 ± 0.2 | 50.0 ± 0.6 | 31.9 ± 0.4 |
| PBT/PLO15(90/10) | 222.4 ± 0.5 | 188.9 ± 0.5 | 50.1 ± 3.6 | 31.1 ± 2.2 |
| PBT/PLO20(95/5) | 223.4 ± 0.5 | 191.4 ± 1.0 | 50.5 ± 2.9 | 33.1 ± 1.9 |
| PBT/PLO20(92.5/7.5) | 222.3 ± 0.0 | 189.5 ± 0.1 | 51.0 ± 1.2 | 32.5 ± 0.8 |
| PBT/PLO20(90/10) | 221.5 ± 0.2 | 188.5 ± 0.3 | 53.7 ± 2.7 | 33.3 ± 1.7 |
